# Pseudoendocrine Sarcoma: Unusual Manifestation in the Lower Leg

**DOI:** 10.7759/cureus.103833

**Published:** 2026-02-18

**Authors:** Torsten Hansen, Mathias Esser, Reiner Wirbel, Cornelia S. L Müller, Abbas Agaimy

**Affiliations:** 1 Pathology, Medical Service Center for Histology, Cytology, and Molecular Pathology Trier GmbH, Trier, DEU; 2 Orthopedic Surgery, Verbundkrankenhaus Wittlich, Wittlich, DEU; 3 Dermatopathology, Medical Service Center for Histology, Cytology, and Molecular Pathology Trier GmbH, Trier, DEU; 4 Pathology, University Hospital Erlangen, Friedrich-Alexander University of Erlangen-Nürnberg (FAU), Erlangen, DEU

**Keywords:** beta-catenin, cd56, ctnnb1 mutation, differential diagnosis, immunohistochemistry, molecular pathology, neuroendocrine-like, pitfall, pseudoendocrine sarcoma, s100

## Abstract

Pseudoendocrine sarcoma is a recently described rare soft tissue neoplasm. The tumor predominantly affects older adults, with the paravertebral soft tissue as a predilection site. We report on an unusual location of pseudoendocrine sarcoma in the right gastrocnemius muscle of a 59-year-old male patient. Histology showed a remarkable neuroendocrine-like morphology. Immunohistochemically, the tumor cells were negative for chromogranin, synaptophysin, and INSM-1 but positive for beta-catenin, CD56, and multifocally for S100. By molecular analysis, an S37F point mutation in exon 3 of the CTNNB1 gene was detected. This case, to our knowledge, illustrates a hitherto unreported location of a pseudoendocrine sarcoma in the lower leg. The relevant differential diagnoses are discussed with special regard to CD56 and S100.

## Introduction

Pseudoendocrine sarcoma is a unique, recently described rare soft tissue neoplasm histopathologically highly reminiscent of neuroendocrine neoplasms but lacking true neuroendocrine differentiation (hence the term “pseudoendocrine”). So far, there are fewer than 50 reported cases in the English medical literature, including two series of 23 and 12 patients, respectively, as well as several case reports [1-4]. Based on the previous findings, this tumor presents as a deep-seated subfascial soft tissue tumor in older adult patients (median sixth to seventh decade) with a slight male predominance. The paravertebral region is described as a major predilected anatomic site [2-4]. Despite its neuroendocrine-like growth pattern and cellular features, the tumor immunohistochemically lacks neuroendocrine markers, including chromogranin, synaptophysin, and INSM-1, as well as different types of keratins. However, pseudoendocrine sarcoma is positive for beta-catenin in > 95% of cases and typically for CD56, too [2,3]. The beta-catenin expression as a sign of Wnt signaling activation is due to underlying CTNNB1 point mutations [4]. The detection of these hotspot mutations has been demonstrated as a useful method in the confirmation of the diagnosis. In contrast to its deceptively bland morphology, this neoplasm has a propensity for destructive local recurrences in about 40% and for metastasis to the lung (up to 20%) and, less often, to lymph nodes and liver [2,3]. Thus, pseudoendocrine sarcoma is designated as an intermediate-grade sarcoma in the upcoming 6th edition of the World Health Organization Classification of Bone and Soft Tissue Tumours [5]. In this case report, we present a patient with pseudoendocrine sarcoma in an unusual anatomic site and discuss the relevant findings in comparison with the previous literature. In particular, we highlight key morphologic and immunophenotypic pitfalls.

## Case presentation

**Figure 1:**
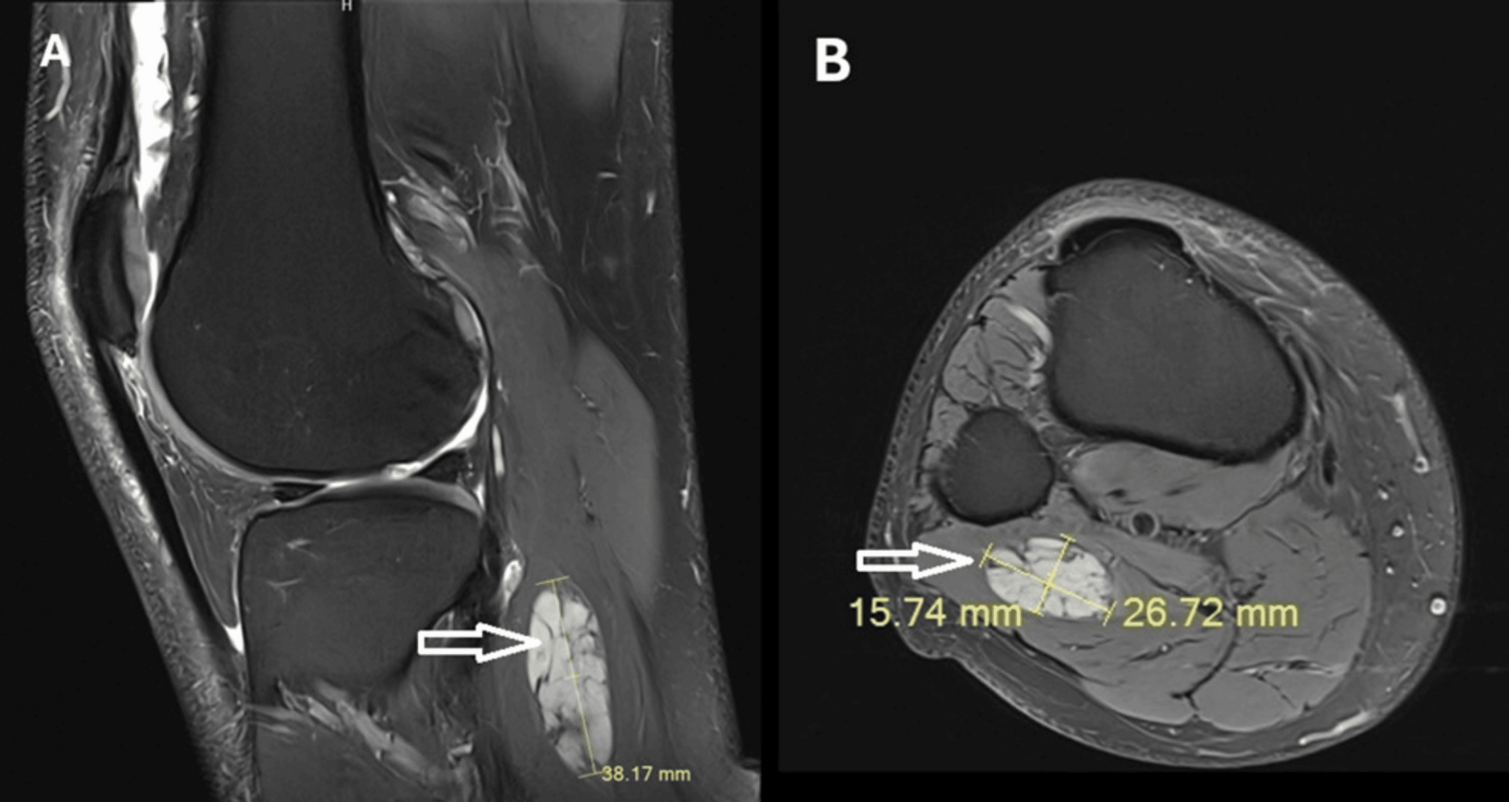
Magnetic resonance imaging (MRI) A: sagittal, B: axial T2-weighted MRI of the right lower leg demonstrating the tumor mass (arrows) in the gastrocnemius muscle measuring up to 38.17 mm.

A 59-year-old male patient with a lesion of the right medial meniscus underwent MRI scanning of the right knee. Incidentally, the analysis revealed a sharply demarcated nodule located in the right gastrocnemius muscle (Figure 1) measuring 38.17 mm at max. The tumor mass was surgically removed. For further histopathological and molecular diagnosis, the tissue specimen was fixed in 4% buffered formaldehyde, paraffin-embedded, and processed according to routine standard protocols.

**Figure 2:**
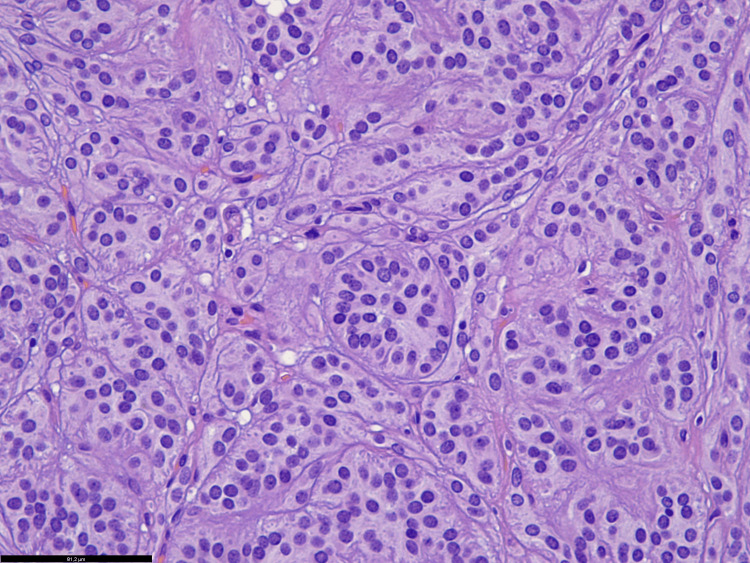
Histology Detailed histological image of the tumor revealing nests and trabeculae of epithelioid cells with eosinophilic cytoplasm and round nuclei with granular chromatin (HE, bar = 81 µm).

Histology revealed a well-delineated, lobular, circumscribed intramuscular tumor with a fibrous pseudocapsule. The epithelioid tumor cells were arranged in nests, sheets, and trabeculae. They showed eosinophilic cytoplasm and round to ovoid nuclei with granular chromatin, but without prominent nucleoli (Figure 2). There was only a little pleomorphism, the mitotic activity was low, and necroses were not found. The tumor was well vascularized and showed variable amounts of stromal hyalinization.

**Figure 3:**
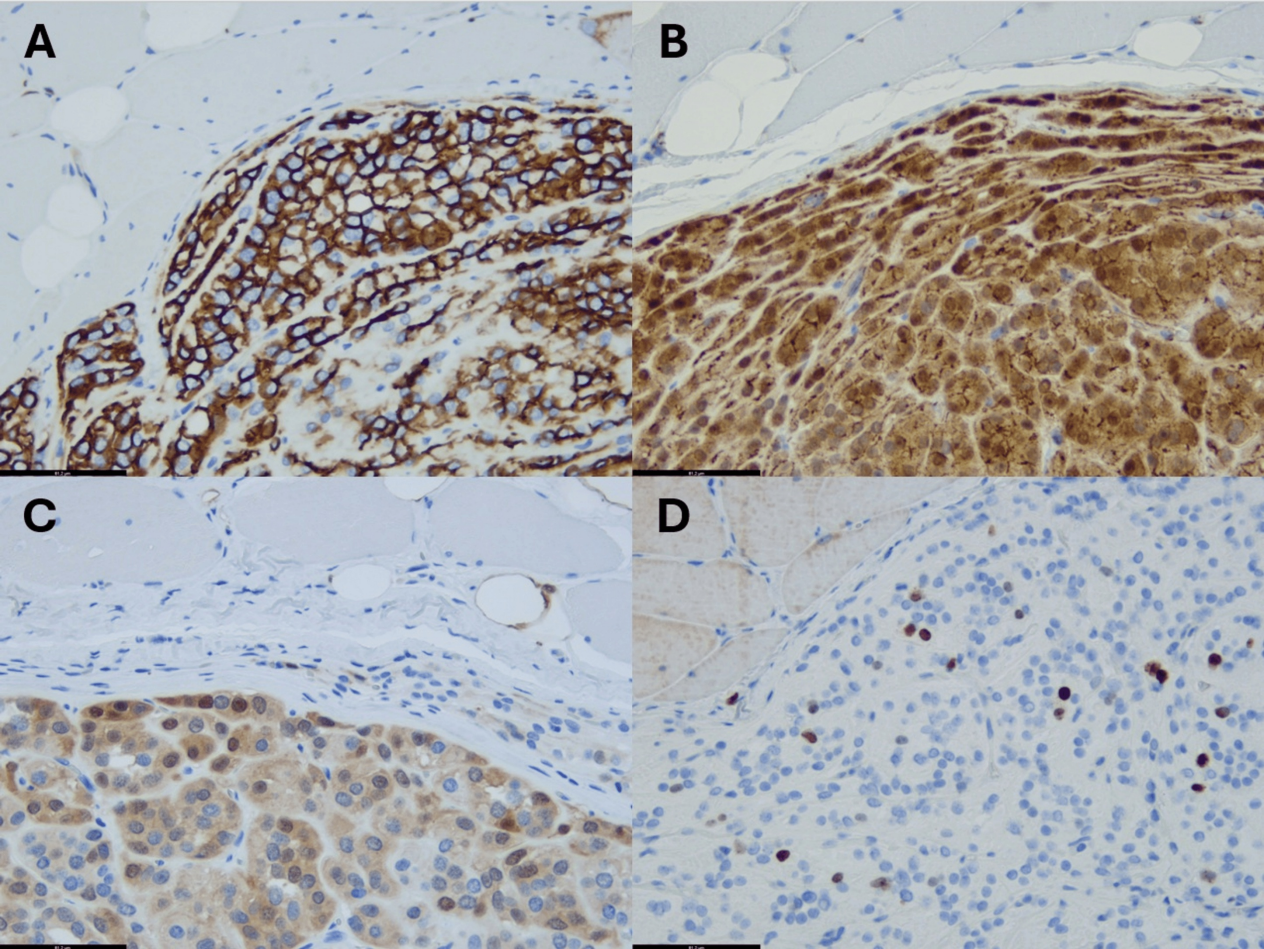
Immunohistochemistry Representative images of immunohistochemistry. A: strong expression of CD56; B: diffuse cytoplasmic and nuclear positivity of beta-catenin; C: multifocal positivity of S100; D: Ki67 labeling (each bar = 81 µm).

Due to its strong resemblance to neuroendocrine or paraganglioma-like neoplasia, we first performed immunohistochemistry with antibodies against cytokeratin, synaptophysin, chromogranin, and INSM-1, all revealing negative labeling. However, CD56 was strongly positive, and labeling for S100 revealed a multifocal labeling (Figure 3), while SOX-10, microphthalmia-associated transcription factor (MITF), HMB-45, desmin, myogenin, and smooth-muscle-specific actin were negative. In addition, the tumor cells were partially positive for CD34, and they especially showed a diffuse cytoplasmic and nuclear positivity for beta-catenin (Figure 3). Ki67 was positive in up to 3%. Performing molecular pathology, we identified a point mutation of CTNNB1 in exon 3 (c. 110C>T, p. Ser37Phe). In summary, these findings were highly suggestive of a pseudoendocrine sarcoma. Due to the paucity of this entity, the case was sent to a reference pathologist in soft tissue pathology for consultation (Abbas Agaimy), rendering the final diagnosis of a pseudoendocrine sarcoma.

Following the recommendations of the reference pathologist, additional radiological analysis was performed because of the unusual location of the primary tumor in the knee region. However, further MRI (particularly of the spine area) did not reveal any pathological findings. The tumor was removed by closed margins (R0 narrow). A short follow-up of six months demonstrated no evidence for local recurrence or metastasis.

## Discussion

In this case study, we report on an unusual case of pseudoendocrine sarcoma, a recently described soft tissue tumor that will be included in the upcoming 6th edition of the World Health Organization Classification of Bone and Soft Tissue Tumours [5]. While the male sex and the age of our patient are mainly in line with the hitherto described clinical findings, the anatomical site presented herein is unusual. Based on the previous literature, the paravertebral region is the typical predilection site for this neoplasm. In the series of Papke et al. [2] and Fernandez-Perez et al. [3], 65% and 91.5%, respectively, arose in paravertebral/paraspinal soft tissue. There are some other rare locations, such as the posterior head [2,6], orbit [2], neck [7], chest wall [8], thigh [2,3], and inguinal region (for a recent summary, see also Fernandez-Perez [3]), while an occurrence in the gastrocnemius muscle, i.e., the lower leg, to our knowledge, has not been reported yet. Since radiological analysis (especially MRI of the spine) did not reveal any suspicious tumor mass in our presented patient, we conclude that the lower leg is the primary site of the tumor. This finding contributes to the view that, infrequently, the occurrence of pseudoendocrine sarcoma in a location other than the truncal soft tissue is possible.

Concerning the histopathological and immunohistochemical findings as well as molecular pathology analysis, our data are mainly in agreement with previous reports. According to previous studies, the differential diagnoses include a large number of potential pitfalls, including neuroendocrine tumor, pseudopapillary tumor of the pancreas, glomus tumor, paraganglioma, and meningiomas. In cases with prominent myxoid stroma, mesenchymal tumors with chondromyxoid features should be additionally considered [1,8]. It is generally known that pseudoendocrine sarcoma is negative for the classical neuroendocrine markers chromogranin, synaptophysin, and INSM-1. However, frequent expression of CD56 was previously mentioned (7/7 cases in the series of Fernandez-Perez et al. [3]), and this profile has been found in our case, too. Together with the remarkable morphology, this may lead to the misinterpretation of a neuroendocrine tumor. This potential pitfall must be taken into mind, since CD56 is ubiquitously expressed in both neuroendocrine and non-neuroendocrine neoplasms, limiting its diagnostic utility [3].

The same holds true regarding the immunohistochemical labelling of S100. In our case, we found a multifocal positivity of S100, which has been described by 41-55% of the previous cases. Since malignant melanomas typically show positivity of S100 and may exhibit neuroendocrine differentiation, even by means of immunohistochemistry [9,10]. Although rare, this differential diagnosis is of particular relevance in routine dermatopathological practice. Therefore, we recommend the use of additional melanoma markers such as HMB45, Melan-A, SOX-10, or MITF. By applying SOX-10, MITF, and HMB-45, we could exclude malignant melanoma in our case.

Molecular analysis is of major importance for the diagnosis of pseudoendocrine sarcoma. So far, hotspot mutations in exon 3 of the CTNNB1 gene have been found, mainly in codon 33, followed by codons 37 and 32. The S37F point mutation being identified in our patient was obtained in 4/11 cases in the series of Fernandez-Perez et al. [3], and it has been mentioned by Papke et al. [2] as well. Activating CTNNB1 mutations are observed in a variety of tumors across different histological subtypes. In brief, these mutations contribute to the accumulation and nuclear translocation of the beta-catenin protein, thereby activating the Wnt signaling pathway [3,11]. Further molecular investigation is needed for potential therapeutic development in pseudoendocrine sarcoma patients by targeting this pathway [3]. So far, primary surgical resection is the treatment of choice. However, this sarcoma leads to recurrences and metastases in a significant percentage; therefore, adjuvant therapy modalities are necessary, especially since chemotherapy and/or radiotherapy are controversial. Moreover, a long-term and close surveillance of pseudoendocrine sarcoma is recommended by the fact that the recurrences may occur even decades after the primary diagnosis [3]. From that view, our case report has a significant limitation regarding the comparatively short follow-up time of only six months.

## Conclusions

We present an unusual case of a pseudoendocrine sarcoma, which is a recently described intermediate-grade sarcoma. While generally presenting with a strong predilection for the paravertebral soft tissue, other infrequent locations are possible, such as the lower leg, as reported herein, to our knowledge, for the first time. Concerning the differential diagnoses, the diffuse positivity of CD56 as a typical feature of pseudoendocrine tumor is a potential pitfall regarding neuroendocrine tumors. On the other hand, multifocal positivity of S100 should not be misinterpreted as malignant melanoma, especially since the latter might display neuroendocrine differentiation. Thus, immunohistochemical assessment should include a panel of both neuroendocrine (in particular, chromogranin, synaptophysin, and INSM-1) and melanocytic markers (e.g., HMB45, Melan-A, SOX-10, or MITF). Together with the distinctive nuclear positivity for beta-catenin and molecular detection of hotspot point mutations in the CTNNB1 gene, a final valid diagnosis of this unique tumor can be made.
